# Public Knowledge, Attitudes, and Perceptions of Antimicrobial Resistance in Brazil: Insights from a Nationwide Online Survey

**DOI:** 10.3390/antibiotics15060624

**Published:** 2026-06-20

**Authors:** Victória Ribeiro Silvestre, Gustavo Guimarães Fernandes Viana, Isha Agrawal, Andréia Gonçalves Arruda, Gabriel Augusto Marques Rossi, Carlo Spanu, Fábio Sossai Possebon, Juliano Gonçalves Pereira

**Affiliations:** 1 Department of Animal Production and Preventive Veterinary Medicine, School of Veterinary Medicine and Animal Science, São Paulo State University (UNESP), Botucatu 18618-681, SP, Brazil; victoria.silvestre@unesp.br (V.R.S.); ggf.viana@unesp.br (G.G.F.V.); fabio.possebon@unesp.br (F.S.P.); 2 Department of Veterinary Preventive Medicine, The Ohio State University, Columbus, OH 43210, USA; agrawal.335@buckeyemail.osu.edu (I.A.); arruda.13@osu.edu (A.G.A.); 3 Department of Veterinary Medicine, University Vila Velha (UVV), Av. Comissário José Dantas de Melo, n.21, Vila Velha 29102-920, ES, Brazil; gabriel.rossi@uvv.br; 4 Department of Veterinary Medicine, University of Sassari (UNISS), 07100 Sassari, Italy; cspanu@uniss.it

**Keywords:** antibiotic-free, behavior, health education, one health, survey

## Abstract

**Background:** Antimicrobial resistance (AMR) poses an escalating threat to global health, agriculture, and the environment, demanding urgent multisectoral action under the One Health framework. Despite global awareness efforts, understanding of AMR among the general population remains insufficient, particularly in low- and middle-income countries such as Brazil. This study aimed to evaluate the knowledge, attitudes, and perceptions (KAP) of the Brazilian population regarding AMR. **Methods:** An online questionnaire was distributed through social media platforms between April and August 2025, resulting in 945 valid responses after data cleaning. Quasi-Poisson models were applied to identify demographic predictors of KAP scores while logistic regression models were used to assess the association between KAP scores and antibiotic use-related practices. **Results:** Education level was the strongest predictor of higher KAP scores, whereas age and gender showed inconsistent influence. Only 40.3% of respondents correctly identified antibiotics among commonly used medicines, and 25.9% reported proper disposal of antibiotic packaging. More than half (54.2%) were willing to pay more for antibiotic-free products, although only 26.7% had ever noticed such labeling. Network analysis of open-ended responses indicated that concerns about potential health risks and AMR awareness were the primary motivators for purchasing antibiotic-free products. **Conclusions:** These findings reveal significant gaps in public understanding of antibiotic use and resistance in Brazil, emphasizing the urgent need for targeted educational initiatives, improved public communication, and behavioral interventions to support antimicrobial stewardship and sustainable antibiotic use.

## 1. Introduction

Antimicrobial resistance (AMR) is a complex global challenge affecting human and animal health, agriculture, and the environment, and is recognized as a critical One Health issue [1]. In 2021, AMR was directly responsible for an estimated 1.14 million deaths and associated with 4.71 million worldwide [2]. Beyond its health impacts, AMR imposes substantial economic burdens, with global healthcare costs projected to increase by USD 300 billion to more than USD 1 trillion per year by 2050, disproportionately affecting low- and middle-income countries such as Brazil [3,4].

Although AMR is a global concern, national strategies must be adapted to specific cultural and socioeconomic contexts [5]. The countries that comprise BRICS (originally Brazil, Russia, India, China, and South Africa, with additional members joining subsequently) remain among those with the highest rates of antimicrobial use globally [6]. Compounding this situation, antimicrobial consumption in Brazil increased by around 30% between 2014 and 2019 [7]. To address this persistent and growing issue, the National Plan for the Prevention and Control of Antimicrobial Resistance within the One Health framework was established in Brazil [8]. Although Brazil has prohibited over-the-counter sale of antimicrobials since 2010 [9], in reality this practice continues to persist, particularly in less developed and rural regions [10,11,12].

In food animal production, antimicrobials are still used to promote growth and for therapeutic and prophylactic purposes, especially in low- and middle-income countries [13]. Within the One Health framework, consumer behavior can influence antibiotic use practices in food production. Heightened awareness of AMR has led to more antibiotic-free products becoming available [14], and by choosing to buy them, consumers can pressure the food industry to lower its reliance on antibiotics [15]. This mechanism, however, depends on public understanding, since a major challenge is consumers’ limited grasp of the significance of these products and of the factors justifying their typically higher price [16]. Awareness of antibiotic-free labeling and willingness to pay more for such products therefore constitute behavioral expressions of AMR awareness, linking individual knowledge and perceptions to consumption choices that bear on antimicrobial stewardship.

Human behavior related to antibiotic use plays a decisive role in the emergence and spread of AMR [17,18]. While scientific research on AMR has expanded considerably, effectively translating this knowledge to the general population remains a key challenge [19,20]. In this regard, survey-based studies are valuable tools for identifying gaps in public KAP, thereby helping to define critical points for targeted educational and policy interventions to support antimicrobial stewardship.

To the authors’ knowledge, no large-scale study has yet characterized AMR-related KAP among the Brazilian general public or examined how it relates to sociodemographic characteristics and antibiotic-related behaviors. In addition, the motivations driving consumer interest in antibiotic-free products, a growing but still poorly understood segment in Brazil, remain unexplored.

The present study aimed to assess the Brazilian general public’s KAP regarding AMR through an online survey. Specifically, we examined the associations between KAP scores and both sociodemographic characteristics and antimicrobial use-related behaviors, and explored, through network analysis of open-ended responses, the motivations underlying interest in antibiotic-free products, in order to identify key knowledge gaps and behavioral patterns that may inform targeted strategies to address AMR.

## 2. Materials and Methods

### 2.1. Sampling

A cross-sectional study was conducted using a digital questionnaire in Portuguese administered through Google Forms. Using a non-probability convenience sampling approach, the survey link was disseminated via Facebook, Instagram, and WhatsApp groups. The questionnaire remained open from April to August 2025. Participation in the survey was voluntary and anonymous, no incentives were offered, and respondents were encouraged to share it to broaden reach beyond the authors’ immediate networks. This recruitment strategy was chosen for its broad reach and feasibility in accessing a geographically dispersed population at low cost. It is, however, recognized that convenience sampling through social media tends to over-represent younger, digitally connected, and more highly educated individuals. As the convenience sampling design precludes probabilistic representativeness, demographic characteristics (education, age, gender, and region) were incorporated as covariates in the multivariable analyses to partially account for differences in sample composition when estimating associations. The questionnaire was developed by the research team, which includes researchers with expertise in veterinary public health, epidemiology, and antimicrobial resistance. The ideal response for each item was defined a priori by consensus among the authors based on established AMR knowledge. Before dissemination, the instrument was reviewed internally by the author team for content relevance and clarity of wording. Lastly, the instrument was developed for this study and was not formally pilot-tested or externally validated, so wording may have influenced respondents’ choice of answer, and exact translation to English may also differ slightly in meaning.

### 2.2. Survey Instrument

The structured questionnaire was composed of a mix of open- and closed-ended questions to comprehensively assess the Brazilian population’s KAP regarding AMR. It included variables on demography, knowledge, attitudes, perceptions, and practices related to antimicrobials. Demographic variables were included to evaluate their potential influence on the KAP attributes. The specific questions corresponding to each category are presented in Table 1. Items were assigned to the knowledge, attitude, and perception domains based on their primary content. Knowledge items captured factual recognition and understanding. Attitude items captured evaluative or dispositional stances toward antibiotic use, access, and related choices. Perception items captured beliefs about risk and transmission. Additional items that did not fall within these categories were analyzed and reported separately. The complete translated questionnaire from Portuguese to English is available in Supplementary Material File S1.

### 2.3. Data Analysis

#### 2.3.1. Data Management and Cleaning

Google Forms data were exported to Excel (.xlsx) for cleaning and analysis. A total of 965 responses were recorded. Responses from non-consenting participants, individuals outside Brazil, and those under 18 years were excluded, resulting in 945 valid responses. Questionnaires with incomplete responses, including missing demographic information, were excluded during data cleaning, and all analyses were therefore conducted on complete cases. No imputation of missing values was performed. For KAP and practice variables, all correct or positive responses were recorded as 1 and all incorrect or negative responses were recorded as 0. Age was recategorized according to categories commonly used in AMR literature (18–30, 31–45, 46–60, >60). Data cleaning and coding were conducted in Microsoft Excel (version 16.70, Microsoft Corporation, Redmond, WA, USA).

#### 2.3.2. Statistical Analysis

All statistical analyses and data visualization were performed in R (RStudio version 2025.05.1+513; RStudio, PBC, Boston, MA, USA).

Descriptive Statistics: Descriptive statistics were computed for variables corresponding to respondents’ demographic and KAP variables and were presented as frequencies (proportions). Cumulative KAP scores were calculated, and summary statistics (mean, standard deviation (SD), median, range) were reported.

Inferential Statistics: Associations between KAP scores (count outcome) and demographic variables were assessed using multivariable Poisson regression. The Poisson regression model was assessed for dispersion using the Pearson chi-square–to–degrees-of-freedom ratio and a simulation-based procedure implemented in the DHARMa R package. In both cases, significant dispersion estimates <1 were obtained, indicating under-dispersion (variance is less than the mean). Therefore, a quasi-Poisson model was fitted and used for inference in the final analysis as it allows estimation of dispersion parameters and adjusts standard errors accordingly, providing a more appropriate inference in under-dispersed data [21]. The main-effects multivariable models included education level, age category, gender, and region as explanatory variables. Variable selection was performed using backward selection, a structured modeling approach starting with a full model composed of variables that are biologically and epidemiologically relevant. Variables were removed sequentially from the main-effects model using a liberal threshold (*p* < 0.20) to avoid prematurely excluding potentially important predictors or confounders [22]. Statistical significance was set at *p* ≤ 0.05.

To further assess whether the association between demographic characteristics and KAP scores varied by education level, interaction analyses were then conducted using quasi-Poisson regression models. For each KAP outcome, separate interaction terms were added between education level and each demographic variable (age category, gender, and region), while retaining the relevant main effects and adjusting for the remaining covariates from the reduced main-effects model. Interaction models were compared with their corresponding reduced main-effects models using F-tests, and interaction terms that improved model fit were retained in the final models. Model-based predicted scores and corresponding 95% confidence intervals (CIs) were estimated from the final interaction models and used to visualize significant interaction effects. For the final interaction models, adjusted incidence rate ratios (IRRs), 95% CIs, and *p*-values were reported for the main effects, whereas interaction effects were presented graphically.

Logistic regression analyses were conducted to assess factors associated with antimicrobial-related practices. First, univariable logistic regression models were used to examine the association between demographic characteristics as independent variables (education level, age category, gender, and region) and selected antimicrobial-related practices as outcomes (willingness to pay more for antibiotic-free food and questioning medical prescriptions following a consultation). Subsequently, multivariable logistic regression models were fitted to evaluate the association between KAP scores as predictor variables and antimicrobial-related practices as outcomes (antimicrobial disposal practices and prescription-following practices). The statistical significance was established at a *p*-value ≤ 0.05 and odds ratios (OR), their 95% CIs, and *p* values were calculated and reported.

The last question of the survey was an open-ended question asking what would motivate the respondent to purchase antibiotic-free products. The qualitative analysis of the question was performed using a co-occurrence network.

Open-ended survey responses regarding participants’ opinions on AMR, originally provided in Portuguese, were translated into English and were analyzed using network text analysis (NTA) to identify emerging themes and interrelationships among concepts. All qualitative responses were aggregated into a single corpus (text data) and preprocessed in R [23]. The corpus was broken down into individual words (tokens), and each token represented a single linguistic unit (unigrams). Additional text processing included lemmatization and the removal of standard English stop-words. A sliding-window co-occurrence matrix was also generated using a window size of four words to capture local semantic context while limiting the inclusion of more distant and potentially unrelated word associations [24]. Two terms were considered to co-occur if they appeared within the same four-word segment, reflecting a close contextual relationship. The co-occurrence data were converted into a weighted edge list, and nodes (unique terms) and edges (co-occurrences) were filtered to retain only relationships appearing at least six times. The edge-frequency threshold of six was selected to balance the retention of meaningful associations against the reduction in excessive network complexity and graph density [24]. A network graph was then constructed using the igraph package, where nodes represented terms and edges represented co-occurrence relationships. To improve interpretability, only the largest connected component was retained. Community detection was performed using the Louvain clustering algorithm to identify clusters of semantically related terms representing emergent themes [25]. Degree was calculated to identify the most influential terms within the network.

### 2.4. Ethics

The study was approved by the Research Ethics Committee of the Faculdade de Medicina de Botucatu, UNESP (CEP-FMB/UNESP) (protocol code 7.462.797; CAAE: 85260224.0.0000.5411) and informed consent was obtained from all subjects involved in the study. Responses from participants under 18 years of age were excluded from the analysis.

## 3. Results

The demographic profile of respondents is presented in Table 2. The KAP scores showed a mean knowledge score of 4.70 (SD = 0.58), with a median of 5.00 and a range from 1.00 to 5.00. The mean attitude score was 8.21 (SD = 1.68), with a median of 9.00 and a range from 2.00 to 10.00. The mean perception score was 3.95 (SD = 0.85), with a median of 4.00 and a range from 1.00 to 5.00.

Key knowledge gaps were observed, including limited awareness of culture and antibiogram testing (27.7% incorrect responses), low recognition of antibiotic-free labeling (73.3%), and poor understanding of appropriate antibiotic use, with only 11.1% providing the ideal response.

**Figure 1:**
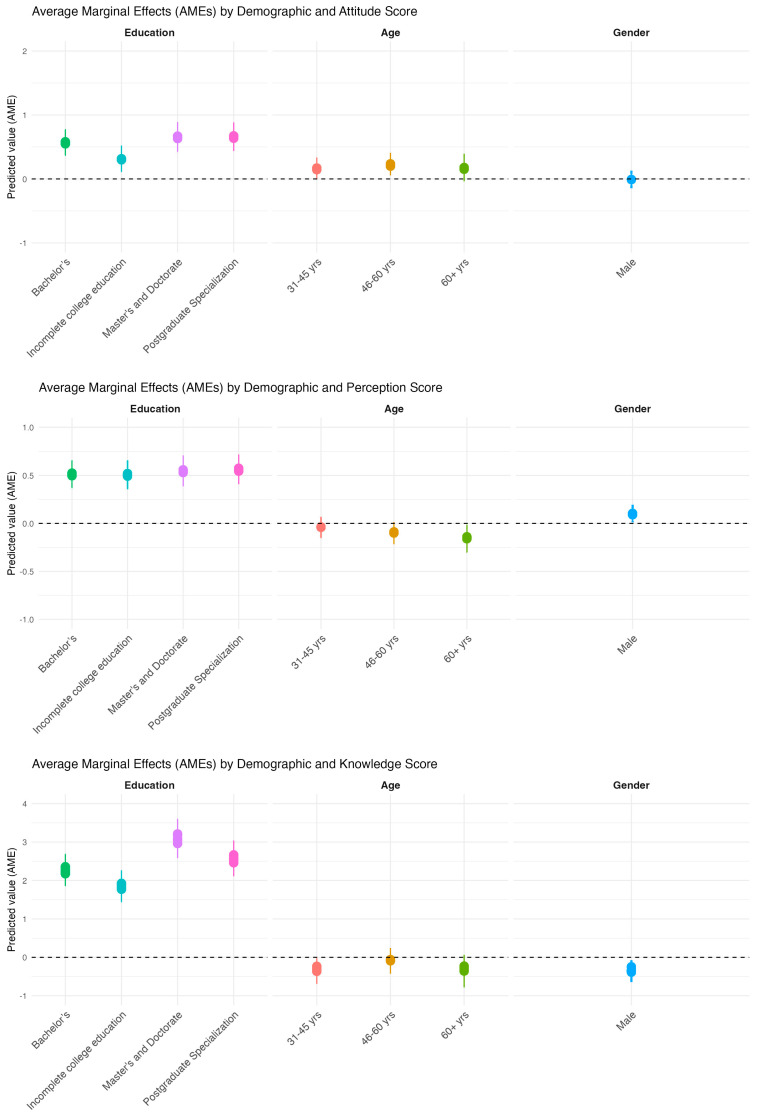
The average marginal effect for the KAP scores in relation to age, education and gender following quasi-Poisson regression models. Each point is the estimated change in the predicted score associated with a given category relative to its reference (less than high school education for education; 18–30 years for age; female for gender). Vertical bars denote 95% CIs. Values above zero (dashed line) indicate higher scores than the reference category, and intervals crossing zero indicate non-significant effects.

Figure 1 shows the average marginal effects for the KAP questions in relation to age, education, and gender. Among the variables examined, education displayed the largest positive marginal effects across the three KAP domains, whereas age and gender showed comparatively smaller effects, with several CIs overlapping zero, suggesting limited or non-significant effects.

Quasi-Poisson regression models examined associations between demographic characteristics and knowledge, perception, and attitude scores, with diagnostics indicating acceptable model fit.

Knowledge model: Relative to respondents with less than a high school education, higher education levels were associated with significantly greater knowledge scores, with IRRs ranging from 1.38 for incomplete college to 1.56 for master’s or doctoral degrees (all *p* < 0.001). Compared with females, males demonstrated slightly higher knowledge scores (IRR = 1.10, 95% CI: 1.01–1.20, *p* = 0.036), although the magnitude of this effect was modest. Age and region showed minimal influence on knowledge scores, with only a small reduction observed among respondents aged 31–45 years and no significant regional differences (Table 3).

**Figure 2:**
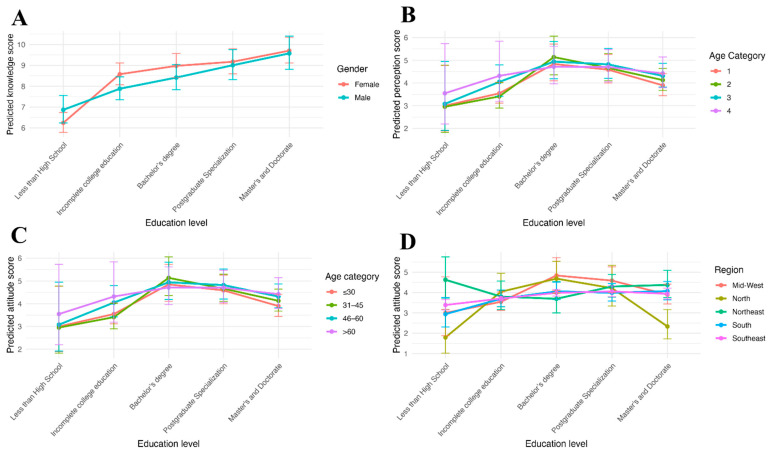
Predicted KAP scores across education levels, stratified by demographic subgroups, from quasi-Poisson interaction models: (**A**) knowledge by gender, (**B**) perception by age category, (**C**) attitude by age category, and (**D**) attitude by region. Points represent model-estimated effects and vertical bars indicate 95% confidence intervals.

A statistically significant interaction emerged between education level and gender, indicating that the positive effect of education on knowledge differed by gender (Figure 2). For males, the educational advantage was attenuated across several education levels. Specifically, the knowledge score among males with incomplete college education was 16% lower than expected from the additive effects alone (IRR = 0.84, 95% CI: 0.75–0.92, *p* < 0.001). Similar reductions were observed for males with a bachelor’s degree (IRR = 0.85, 95% CI: 0.77–0.94, *p* = 0.002) and postgraduate specialization (IRR = 0.89, 95% CI: 0.80–1.00, *p* = 0.040). The interaction for males with a master’s/doctorate approached significance (IRR = 0.90, 95% CI: 0.80–1.00, *p* = 0.055).

Perception model: All higher education levels were associated with significantly higher perception scores compared with less than high school education, with IRRs remaining mostly similar ranging from 1.11 for incomplete college to 1.13 for postgraduate specialization (all *p* < 0.001). Age category showed little association with perception scores after adjusting for other demographic variables in the model. Compared with the youngest age group, respondents aged 31–45 years demonstrated a borderline reduction in perception score (IRR = 0.93, 95% CI: 0.86–1.00, *p* = 0.060), while no significant differences were observed for older age categories. Gender was a significant predictor of perception score. Males had slightly higher perception scores compared with females (IRR = 1.02, 95% CI: 1.01–1.04, *p* = 0.005), although the magnitude of this effect was modest. No statistically significant regional differences in perception score were observed. All regional IRRs were close to unity, indicating minimal geographic variation (Table 3).

The interaction between education and age revealed selective effect modification (Figure 2). While most interaction terms were not statistically significant, respondents aged over 60 years with postgraduate specialization exhibited significantly lower perception scores than expected based on main effects alone (IRR = 0.88, 95% CI: 0.80–0.97, *p* = 0.011). A borderline positive interaction was observed for incomplete college education among respondents aged 31–45 years (IRR = 1.10, 95% CI: 1.00–1.21, *p* = 0.053).

Attitude model: Associations between education and attitude scores were weaker and did not reach statistical significance, the strongest, albeit borderline, estimate being observed among respondents with a bachelor’s degree (IRR = 1.62, 95% CI: 1.02–2.75, *p* = 0.054). Age effects were modest. Only adults over 60 years demonstrated significantly higher scores (IRR = 1.18, 95% CI: 1.04–1.34). Regionally, participants from the North had significantly lower scores relative to the reference region (IRR = 0.60, 95% CI: 0.43–0.82). Gender was not associated with attitude scores (Table 3).

For the interaction between education and age, most individual interaction terms were non-significant, although the effect of holding a bachelor’s degree was attenuated among adults over 60 years (IRR = 0.82, 95% CI: 0.71–0.96).

For the interaction between education and region, several individual interaction terms were significant, indicating notable regional variation in the effect of education. In the North, incomplete college education (IRR = 1.90), bachelor’s degree (IRR = 1.62), and postgraduate specialization (IRR = 1.54) were associated with increased attitude scores than would be expected based on the independent main effects of education and region alone. Conversely, in the Northeast, the effect of a bachelor’s degree was reversed (IRR = 0.49, 95% CI: 0.27–0.86), indicating substantially lower scores for this subgroup.

Across all models, education consistently emerged as the strongest determinant of KAP toward AMR, whereas the effects of age and gender were small and inconsistent (Table 3).

Table 4 summarizes logistic regression results examining associations between KAP scores and antimicrobial practices.

### Predictors of Appropriate Antibiotic-Related Practices

For “disposal practices”, both knowledge and attitude scores were positively associated with appropriate behaviors. Each unit increase in the knowledge score was associated with 1.13 times higher odds of indicating a correct antimicrobial disposal (95% CI = 1.02–1.25; *p* = 0.016). Similarly, a unit increase in the attitude score corresponded to 1.39 times higher odds of correct antimicrobial disposal practice (95% CI = 1.16–1.68; *p* < 0.001). Perception score showed a negative but borderline significant association (OR = 0.78; 95% CI = 0.61–1.01; *p* = 0.056), suggesting that participants with stronger risk perceptions had slightly lower odds of proper disposal practice, though this effect was not statistically significant.

For “following prescription practices”, a higher attitude score was significantly associated with adherence to prescribed antimicrobial use (OR = 1.50; 95% CI = 1.03–2.19; *p* = 0.036). Knowledge and perception scores were not significantly associated with this outcome (*p* > 0.1).

Overall, attitude toward AMR emerged as the most consistent predictor of responsible antimicrobial practices, whereas knowledge showed a modest effect limited to disposal behavior, and perception demonstrated no clear association across practices.

Univariable logistic regression models examined demographic predictors of respondents’ willingness to pay more for antibiotic-free food. Overall, 54.2% (*n* = 512) indicated they would be willing to pay a higher price for such products. Gender was not significantly associated with willingness to pay more. Participants with incomplete college education had significantly lower odds of willingness to pay more compared with those with a high school education or less (OR = 0.57, 95% CI: 0.36–0.90, *p* = 0.016). Higher education categories exhibited slightly elevated odds, though none reached statistical significance (*p* > 0.05). Respondents from the Northeast had markedly higher odds of willingness to pay more (OR = 4.87, 95% CI: 1.54–18.86, *p* = 0.011), whereas other regions did not differ significantly from the reference. Compared with the youngest age group, older categories had significantly higher odds of willingness to pay more (ORs = 2.07–3.13; all *p* < 0.001).

Univariable logistic regression models were also used to examine demographic predictors of respondents’ questioning medical prescriptions following a consultation. Notably, only 50.2% of respondents indicated this practice. Gender was not significantly associated with this behavior. Education level demonstrated a strong and graded association with questioning medical prescriptions. Compared with respondents with a high school education or less, those with a bachelor’s degree had significantly higher odds of evaluating/debating prescriptions (OR = 2.47, 95% CI: 1.54–4.01, *p* < 0.001). Even stronger associations were observed for individuals with postgraduate specialization (OR = 3.01, 95% CI: 1.82–5.07, *p* < 0.001) and those with a master’s or doctoral degree (OR = 3.29, 95% CI: 1.95–5.61, *p* < 0.001). Respondents with incomplete college education showed a nonsignificant increase in odds (OR = 1.39, 95% CI: 0.87–2.26, *p* = 0.18). Geographic region was not a significant predictor. Age was positively associated with prescription evaluation for some categories. Compared with the youngest age group, respondents in age categories 2 (31–45 years) and 3 (46–60 years) had significantly higher odds of evaluating or debating prescriptions (31–45 years: OR = 1.83, 95% CI: 1.31–2.55, *p* < 0.001; 46–60 years: OR = 1.70, 95% CI: 1.21–2.40, *p* = 0.002).

**Figure 3:**
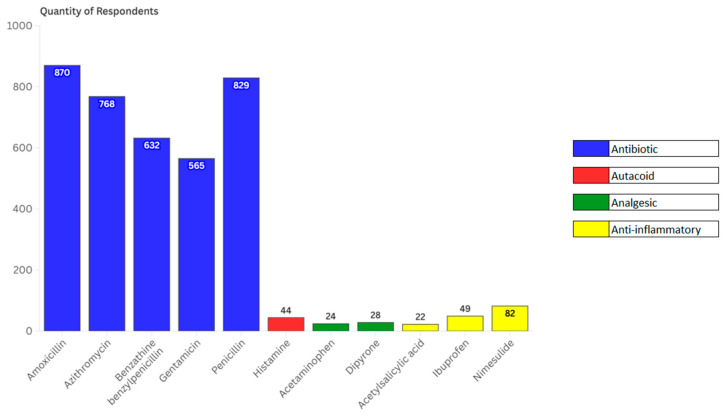
Frequency of respondents who identified each medicine as an antibiotic. The medicines listed in the questionnaire included either the active ingredient name or the commercial brand name.

In the question “Check the options that show brands/actives for antibiotic drugs,” participants were presented with 11 medicines commonly used in Brazil. Respondents could select multiple options. A response was only considered correct if all five antibiotics were chosen and no additional, incorrect medicines were selected. Figure 3 presents the descriptive results, showing the number of respondents who identified each medicine as an antibiotic. Although most respondents correctly identified antibiotic drugs, only 381 participants (40.3%) exclusively selected all the antimicrobial agents without including drugs from other therapeutic classes.

**Figure 4:**
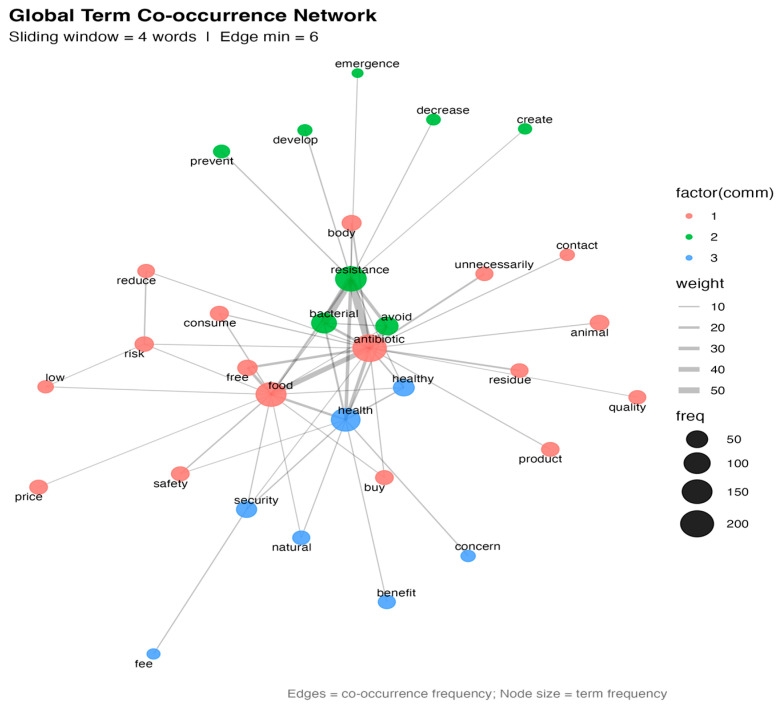
Co-occurrence network of terms derived from respondents’ open-ended answers to the question “What would motivate you to buy an antibiotic-free product?” Each node represents a unique word, with node size proportional to word frequency and edges representing co-occurrence relationships between terms within a four-word context window. The colors denote distinct thematic clusters identified through Louvain community (cluster) detection.

The network analysis of respondents’ open-ended answers to the question “What would motivate you to buy an antibiotic-free product?” revealed distinct thematic clusters that capture the multifaceted nature of consumer motivations (Figure 4). Three main domains were identified. The red cluster was organized around the term “antibiotic” with different sometimes unrelated words; the blue cluster grouped health-related motivations, and the green cluster contained terms more related to resistance itself.

## 4. Discussion

Although AMR is globally recognized as a critical public health priority, particularly in high-income countries, its importance in Brazil does not yet appear to be fully reflected in national awareness or prioritization efforts [26]. Developing and maintaining comprehensive national programs to address AMR under the One Health framework remains a major challenge, given the wide range of human, animal, and environmental factors involved [27]. To the authors’ knowledge, this study represents the first large-scale assessment of AMR-related KAP among the Brazilian general public, distinct from a previous Brazilian research that analyzed AMR-related KAP among physicians in a Brazilian teaching hospital [28].

Antibiotic-free meat has become increasingly available in recent years [14,29]. However, the finding that only 252 respondents (26.7%) had ever noticed an antibiotic-free label on packaging is striking, suggesting that these products have not yet achieved broad recognition among Brazilian consumers, particularly in less developed regions, where purchasing power tends to be lower. Similarly, only 512 participants (54.2%) indicated willingness to pay more for antibiotic-free products. This limited readiness to invest in such items not only suggests that pricing may be the primary driver for product selection for many respondents, but it also underscores the need for greater public understanding of AMR and the value of antibiotic-free production systems. Previous studies have shown that as public knowledge of AMR increases, willingness to purchase these products also rises [15,30].

It is also notable that only 40.3% of respondents correctly identified antibiotics among a list of commonly used medicines. If the majority of individuals are unable to recognize which drugs are antibiotics, expecting them to comprehend the broader implications of AMR is unrealistic. This knowledge gap may also contribute to inappropriate antibiotic use [31]. This is especially consequential in Brazil, where self-medication with antibiotics remains common [12] and has even increased in some settings, rising from 19.2% to 30.7% among adults in Manaus between 2015 and 2019 [32]. Individuals who cannot recognize which medicines are antibiotics are poorly positioned to identify when antibiotics are being used unnecessarily, to question inappropriate demand, or to recognize their own misuse, allowing such practices to go unnoticed. The relevance of curbing this demand is supported by evidence that restricting over-the-counter antimicrobial sales in São Paulo was followed by reduced resistance to amoxicillin and trimethoprim/sulfamethoxazole, although the effect varied by drug [33]. Improving the public’s ability to identify antibiotics may therefore complement regulatory measures by reducing inappropriate demand and use at its source. Polypharmacy, meaning when an individual uses multiple medications, may further complicate public understanding of antibiotic drug classes [34]. These factors reinforce the role of healthcare professionals in communicating appropriate antibiotic use [35].

A particularly striking pattern in our data is the dissociation between awareness and applied understanding. While 96.4% of respondents reported having heard of antibiotic resistance and 94.5% correctly completed a basic definition of the term, only 11.1% selected the ideal response regarding the appropriate uses of antibiotics and just 40.3% reliably distinguished antibiotics from other classes of medicines. This indicates a clear gap in health literacy, which extends beyond the basic capacity to obtain health information to include the ability to critically appraise and apply it in everyday decisions [36]. Our findings suggest that, for a part of the Brazilian public, knowledge of AMR remains predominantly functional and superficial rather than critical and actionable.

Among all demographic variables, education emerged as the most significant factor, showing a positive association with KAP scores. Similar trends have been documented elsewhere: Muflih et al. [37] reported that higher education levels were associated with greater awareness of AMR, while an African systematic review also identified a consistent positive relationship between educational attainment and AMR knowledge [38]. Comparable patterns have also been observed in general population surveys conducted in Bangladesh [39], Thailand [19], and Japan [40], where higher education was associated with greater AMR awareness and more appropriate antibiotic-related behaviors. Notably, this educational advantage was not entirely uniform across subgroups: interaction analyses indicated that the gradient was steeper among females than males for knowledge, and was somewhat attenuated among older adults for perception and attitude (Figure 2), suggesting that the influence of education on AMR-related KAP may be modulated by gender and age.

Conversely, the influence of gender appears less consistent across studies. Simegn and Moges [20] reported an association between male gender and higher AMR knowledge, whereas Tangcharoensathien et al. [19] found greater AMR awareness among females. Alternatively, Azim et al. [39] reported no significant gender-related differences in KAP outcomes. In the present study, gender showed limited but statistically significant associations with AMR KAP, primarily affecting perception scores, suggesting a context-dependent or secondary influence compared with education, making gender a weaker target for population-level interventions.

Age-related effects have also varied across studies. Guo et al. [41] identified strong associations between age, knowledge deficits, and inappropriate antibiotic practices among younger and middle-aged adults, while Sindato et al. [42] reported a positive correlation between increasing age and higher knowledge, attitudes, and practices scores related to antimicrobial use and resistance. In contrast, the present study observed mostly modest and inconsistent associations between age and KAP domains, suggesting that age alone may not be a primary determinant of AMR-related understanding and behaviors. This lack of a clear age gradient argues against age-targeted campaigns and reinforces education as the more appropriate focus for intervention.

Only 245 respondents (25.9%) reported correctly disposing of antibiotic packaging. As shown in Table 4, this behavior was positively associated with higher knowledge and attitude scores. Perkola et al. [43] showed how plastic tablet containers can harbor different medicine residues, which could also be the case for antibiotic packaging; were this to occur, residues reaching the environment could contribute to the selective pressure associated with antimicrobial resistance. The contribution of discarded packaging to this process has not been quantified and is likely small relative to that of unused or excreted antibiotics. Nonetheless, correct disposal of antibiotic-related materials remains a simple, avoidable measure, and informational policies promoting it could help limit environmental dissemination of antimicrobial residues in Brazil. Notably, perception scores were not associated with proper disposal, in contrast to knowledge and attitude scores. This indicates that perceiving antibiotic resistance as a risk does not, by itself, translate into appropriate disposal behavior, which may instead depend on practical knowledge and on familiarity with adequate disposal channels.

Adherence to medical prescriptions also demonstrated a strong positive association with higher attitude scores. This relationship suggests that individuals who exhibit more evaluative and responsible attitudes toward behaviors and policies are more likely to follow medical guidance properly. The importance of prescription adherence cannot be overstated, as deviations from prescribed treatments can result in therapeutic failure and foster the development of resistant bacterial strains [44]. More broadly, across both disposal and prescription adherence, attitudes were a more consistent predictor of responsible practices than knowledge, implying that interventions raising knowledge without also shaping attitudes may be insufficient to change behavior.

Overall, age and region showed the strongest associations with willingness to pay more for antibiotic-free products, while gender had no significant effect and education demonstrated only one meaningful association. Regional differences may reflect broader socioeconomic and structural factors, including income and access to information across geographic areas; however, given the small number of respondents from regions other than the Southeast, these regional associations should be viewed with caution. The concentration of effects in age and region, rather than across all demographic variables, suggests that willingness to pay for antibiotic-free products may be patterned by specific contextual factors rather than reflecting a broad, uniform engagement with AMR, pointing to selective rather than population-wide drivers of this behavior.

Regarding questioning medical prescriptions, education emerged as the strongest and most consistent predictor, with higher levels of educational attainment associated with markedly increased odds of this behavior. Age showed moderate positive associations, while gender and region were not significant predictors. These results indicate that critical engagement with prescribed treatments may be influenced more by educational and experiential factors than by demographic characteristics such as gender or geographic location.

The co-occurrence network analysis of respondents’ motivation to purchase antibiotic-free products revealed distinct clusters of answers. The blue cluster was primarily associated with health-related concerns, with frequent terms such as “health”, “healthy”, and “security”, indicating that personal and family health protection represents the principal motivation for purchasing antibiotic-free products. The green cluster consisted of more tightly connected nodes containing terms such as “resistance”, “bacterial”, and “avoid”, suggesting that awareness of AMR itself served as the main driver within this group. In contrast, the red cluster was more diffuse and spatially dispersed throughout the network. Although its most prevalent terms were “antibiotic” and “food”, the red cluster did not display a coherent thematic focus comparable to the blue or green groups. Instead, it reflected a heterogeneous set of loosely related motivations.

Overall, the network demonstrated that consumer motivations center predominantly on health protection (blue cluster) and concern about AMR (green cluster), accompanied by a third, less structured category (red cluster) representing diverse individual motivations. These structural patterns suggest that public interest in antibiotic-free products is inherently multidimensional, encompassing both self-protective and socially conscious perspectives. These results indicate that interest in antibiotic-free products does not reflect a single reason but several coexisting motivations, with the diffuse red cluster, consistent with the limited label recognition (26.7%) and modest willingness to pay more (54.2%) reported above, likely representing consumers without a clearly articulated reason, for whom strengthening AMR-related understanding is a concrete target for educational and labeling interventions. From a communication standpoint, this segmentation suggests that a single, uniform message is unlikely to reach all consumers. Health-framed messaging may resonate most with the health-oriented cluster, resistance-focused messaging can reinforce and expand the AMR-aware segment, and foundational AMR awareness-building is likely needed for the diffuse group that lacks a clear rationale. To the authors’ knowledge, this is the first study to explore, through an open-ended question, the motivations that drive the Brazilian general public to purchase antibiotic-free products.

This study contains limitations. Although it gathered responses from individuals with diverse demographic and geographic backgrounds, the use of a non-random convenience sampling strategy and the predominance of survey dissemination within an academic environment likely contributed to a higher average educational level among participants compared with the general Brazilian population. Notably, 60.9% of respondents had completed higher education, compared with 18.4% of the Brazilian population aged 25 or older according to national data [45]. Because educational attainment was the strongest predictor of KAP scores in our models, this over-representation of more highly educated respondents most likely caused the descriptive estimates to overstate the level of AMR-related knowledge, attitudes, and perceptions relative to the general population. Consequently, the knowledge gaps identified in this study may be even more pronounced nationwide, and the absolute KAP estimates should be regarded as upper-bound approximations rather than nationally representative values. Furthermore, the sample was also predominantly female (71.4%) and most respondents (83.6%) resided in the Southeast region of Brazil, which coincides with the location of the university for most authors and the researchers’ main sphere of outreach. The remaining macro-regions were represented by comparatively few respondents; consequently, the regional comparisons reported rest on limited subgroup sizes and may yield imprecise estimates, as reflected in their wide CIs. These regional differences should be interpreted with particular caution and regarded as exploratory rather than definitive. Additionally, there is an absence of income information in the demographic profile section of the questionnaire. Considering the heterogeneity of income in Brazil, this information could have led to another interesting analysis. Furthermore, no external content validation or pilot testing was performed for the questionnaire before administration. We acknowledge that the KAP domains are not mutually exclusive, which is a recognized challenge in KAP research, where standardized instruments are lacking [46]. For example, the willingness-to-pay and prescription-questioning items, while treated here as attitudinal dispositions, border on behavioral intentions and practices and could be classified differently. Their inclusion in the attitude score should therefore be interpreted with this caveat in mind. Lastly, wording may have an impact on respondents’ choice of answer and exact translation to English may also be slightly different in meaning. These factors may limit the generalizability of the findings, and caution should therefore be exercised when extrapolating these results to the broader national context.

Future research should aim to expand the representativeness and depth of AMR perception studies in Brazil. Conducting stratified or regionally balanced surveys could better capture differences in KAP across socioeconomic and geographic contexts. Longitudinal studies assessing how public understanding of AMR evolves over time, particularly in response to national awareness campaigns or policy changes, would also be valuable. Additionally, incorporating qualitative methodologies such as focus groups or semi-structured interviews could provide deeper insights into behavioral drivers and misconceptions that quantitative surveys may overlook. Lastly, future research could also further explore consumer risk perception and trust in food safety systems to better understand how these factors influence attitudes toward antibiotic-free labeling and AMR-related purchasing behaviors.

## 5. Conclusions

This study provides the first large-scale assessment of AMR-related KAP among the Brazilian public, identifying important knowledge gaps as well as limited familiarity with antibiotic-free products. These gaps are likely even wider in the broader population, given that our sample had higher educational attainment and was more concentrated in the Southeast region than the general population. Education level was the main factor associated with higher KAP scores, emphasizing the central role of education in shaping AMR-related knowledge, attitudes, and perceptions. The low recognition of antibiotic-free product labels and the limited willingness to pay more for such products observed in this sample indicate limited familiarity with AMR-related labeling itself, and suggest that its potential value for health and sustainability is not yet widely recognized among Brazilian consumers. Furthermore, consumer motivations for antibiotic-free products were primarily health-related, with some concern for AMR itself. Our study highlights the need for national and regional authorities to prioritize structured, population-targeted educational initiatives that move beyond passive information dissemination and actively promote informed decision-making regarding antibiotic use. Promoting behavior-focused public communication on appropriate antibiotic use, strengthening communication by healthcare professionals during prescribing encounters, and improving public guidance on antibiotic disposal represent concrete strategies supported by the present results. As these findings derive from a non-probabilistic, predominantly Southeast-based sample, they should be interpreted as indicative of important patterns rather than as nationally representative estimates. These actions could strengthen public participation in antimicrobial stewardship and support more sustainable antibiotic use within Brazil’s One Health framework.

## Figures and Tables

**Table 1:** Quantity of respondents who selected the ideal answer for each question from the categories knowledge, attitudes and perceptions regarding antimicrobial resistance.

	Knowledge Questions	Ideal Answers *n* (%)
1	Have you heard of superbacteria?	875 (92.6%)
2	Have you heard about antibiotic resistance?	911 (96.4%)
3	Have you ever heard of culture and antibiogram?	683 (72.3%)
4	Complete the phrase “antibiotic resistance is when…”	893 (94.5%)
5	What is culture and antibiogram?	913 (96.6%)
6	What are the appropriate uses of antibiotics?	105 (11.1%)
7	What is the main action expected from an antibiotic?	902 (95.4%)
8	Selling antibiotics without a medical prescription is forbidden in Brazil	872 (92.3%)
9	It’s possible to detect which is the bacteria causing the infection according to the symptoms of a disease	629 (66.6%)
10	Check the options that show brands/actives for antibiotic drugs:	381 (40.3%)
	Attitudes questions	
1	When I have a headache, an antibiotic can help me.	925 (97.9%)
2	Antibiotics should be easier to access, without need of a medical prescription.	919 (97.2%)
3	The treatment with an antibiotic, can be discontinued as soon as the symptoms finish.	905 (95.8%)
4	Would you pay more in a food that is antibiotic-free?	512 (54.2%)
5	Do you question the medical prescription that you receive after a consultation?	474 (50.2%)
	Perception questions	
1	Do you think that a bacteria can be transmitted from a human to an animal and vice-versa?	853 (90.3%)
2	Do you think food can transmit antibiotic resistance bacteria?	808 (85.5%)
3	Antibiotics are all the same	939 (99.4%)
4	Taking antibiotics before any symptom of a disease, can prevent from being affected with it	908 (96.1%)
5	Any antibiotic is adequate to treat any infection	932 (98.6%)

**Table 2:** Demographic profile of the respondents.

Variable	Category	*n* (%)
Age	18–30	449 (47.5%)
	31–45	212 (22.4%)
	46–60	191 (20.2%)
	>60	93 (9.9%)
Gender	Male	265 (28.1%)
	Female	675 (71.4%)
	Prefer not to answer	5 (0.5%)
Region	Southeast	790 (83.6%)
	South	69 (7.3%)
	North	15 (1.6%)
	Northeast	32 (3.4%)
	Mid-West	39 (4.1%)
Education	High school graduate or lower	104 (11.0%)
	Incomplete Higher Education	266 (28.1%)
	Bachelor’s degree	257 (27.2%)
	Postgraduate specialization	170 (18.0%)
	Master’s or Doctorate degree	148 (15.7%)

States from respondents for each region: North: Acre, Amazonas, Pará, Rondônia, Tocantins. Northeast: Bahia, Ceará, Maranhão, Paraíba, Pernambuco, Piauí, Rio Grande do Norte. Mid-West: Distrito Federal, Goiás, Mato Grosso, Mato Grosso do Sul. Southeast: Espírito Santo, Minas Gerais, Rio de Janeiro, São Paulo. South: Paraná, Rio Grande do Sul, Santa Catarina.

**Table 3:** Adjusted Incidence Rate Ratios (IRR) for demographic predictors of ideal response counts in Knowledge, Attitudes and Perception categories from multivariable quasi-Poisson model with interactions. Interaction effects are presented in Figure 2.

Outcome	Predictor	IRR	95% CI	*p*-Value
Knowledge Score	Intercept	6.02	(5.72–6.34)	<0.001
	Education (Ref: Complete High School or less)			
	Incomplete college education	1.38	(1.30–1.46)	<0.001
	Bachelor’s degree	1.44	(1.36–1.52)	<0.001
	Postgraduate Specialization	1.47	(1.39–1.56)	<0.001
	Master’s and Doctorate	1.56	(1.47–1.66)	<0.001
	Age (Ref: 18–30 yrs)			
	31–45 yrs	0.96	(0.93–0.99)	0.036
	46–60 yrs	0.99	(0.96–1.03)	0.579
	>60 yrs	0.96	(0.92–1.01)	0.087
	* Gender (Ref: Female)			
	Male	1.10	(1.01–1.20)	0.036
Perception Score	Intercept	4.24	(4.13–4.36)	<0.001
	Education (Ref: Complete High School or less)			
	Incomplete college education	1.11	(1.06–1.16)	<0.001
	Bachelor’s degree	1.12	(1.07–1.17)	<0.001
	Postgraduate Specialization	1.13	(1.07–1.20)	<0.001
	Master’s and Doctorate	1.11	(1.05–1.17)	<0.001
	* Age (Ref: 18–30 yrs)			
	31–45 yrs	0.93	(0.86–1.00)	0.060
	46–60 yrs	0.97	(0.91–1.02)	0.302
	>60 yrs	1.00	(0.93–1.08)	0.945
	Gender (Ref: Female)			
	Male	1.02	(1.01–1.04)	0.005
Attitude Score	Intercept	3.41	(3.25–3.57)	<0.001
	Education (Ref: Complete High School or less)			
	Incomplete college education	1.18	(0.76–1.98)	0.472
	Bachelor’s degree	1.62	(1.02–2.75)	0.054
	Postgraduate Specialization	1.53	(0.97–2.57)	0.083
	Master’s and Doctorate	1.30	(0.83–2.18)	0.302
	* Age (Ref: 18–30 yrs)			
	31–45 yrs	0.98	(0.86–1.13)	0.791
	46–60 yrs	1.03	(0.93–1.14)	0.623
	>60 yrs	1.18	(1.04–1.34)	0.011
	* Region (Ref: Mid-West)			
	North	0.60	(0.43–0.82)	0.002
	Northeast	1.55	(0.95–2.66)	0.094
	South	0.98	(0.60–1.71)	0.94
	Southeast	1.13	(0.73–1.88)	0.624

* Variable included in a statistically significant interaction with education level. The corresponding interaction effects are presented in Figure 2.

**Table 4:** Multivariable logistic regression models with antimicrobial practices and following prescription as the outcome variable and KAP scores as predictors.

Outcome	Predictor	OR	95% CI	*p*-Value
Model 1				
Disposal practice	Knowledge score	1.13	1.02, 1.25	0.016
	Perception score	0.78	0.61, 1.01	0.056
	Attitude score	1.39	1.16, 1.68	<0.001
Model 2				
Following prescription	Knowledge score	1.15	0.95, 1.37	0.14
	Perception score	0.97	0.53, 1.60	0.904
	Attitude score	1.5	1.03, 2.19	0.036

## Data Availability

The data presented in this study are available on request from the corresponding author due to privacy and ethical restrictions established by the institutional ethics committee and the informed consent process.

## Supplementary Materials

The following supporting information can be downloaded at: https://www.mdpi.com/article/10.3390/antibiotics15060624/s1, File S1: Questionnaire Answers.
